# Slow fertilization of stickleback eggs: the result of sexual conflict?

**DOI:** 10.1186/1472-6785-6-7

**Published:** 2006-05-19

**Authors:** Theo CM Bakker, Marc Zbinden, Joachim G Frommen, Alexander Weiss, Carlo R Largiadèr

**Affiliations:** 1 Institute for Evolutionary Biology and Ecology, University of Bonn, An der Immenburg 1, D-53121 Bonn, Germany; 2 Institut für Klinische Chemie IKC, Inselspital, University of Bern, CH-3010 Bern, Switzerland; 3 Swiss National Science Foundation, Division Biology and Medicine, Wildhainweg 3, P.O. Box 8232, CH-3001 Bern, Switzerland

## Abstract

**Background:**

The fertilization success in sperm competition in externally fertilizing fish depends on number and quality of sperm. The time delay between sequential ejaculations may further influence the outcome of sperm competition. Such a time interval can load the raffle over fertilization if fertilization takes place very fast. Short fertilization times are generally assumed for externally fertilizing fish such as the three-spined stickleback (*Gasterosteus aculeatus*). In this pair-spawning fish, territorial males often try to steal fertilizations in nests of neighbouring males. This sneaking behaviour causes sperm competition. Sneakers will only get a share of paternity when eggs are not fertilized immediately after sperm release. Contrary to males, females may be interested in multiple paternity of their clutch of eggs. There thus may be a sexual conflict over the speed of fertilization.

**Results:**

In this study we used two different *in vitro *fertilization experiments to assess how fast eggs are fertilized in sticklebacks. We show that complete fertilization takes more than 5 min which is atypically long for externally fertilizing fishes.

**Conclusion:**

This result suggests that the time difference does not imply high costs to the second stickleback male to ejaculate. Slow fertilization (and concomitant prolonged longevity of sperm) may be the result of sexual conflict in which females aimed at complete fertilization and/or multiple paternity.

## Background

The reproductive success of males in teleost fish with external fertilization is often influenced by the level of sperm competition, which is assumed to be raffle-based. If the raffle is "fair", the proportion of eggs fertilized by a male will reflect the proportion of its sperm in the competition. In a "loaded raffle", competing spermatozoa have unequal chances of fertilizing an egg. Even when both males invest the same number of sperm, a competitively inferior male may not fertilize the same amount of eggs as a superior one.

Successful reproduction depends on a male's ability to access and defend a female, on the proportion of a male's sperm in a possible competition [1-3], and on ejaculate characteristics of competing males [4]. The latter includes differential fertilization ability due to several factors and their interactions, such as differences in sperm motility, sperm size, velocity, metabolic rate, and differential success in cryptic female choice [*e.g. *[5,6]]. Even though not acting on the sperm level in a strict sense, sequential ejaculation may bring up time as a load of the raffle for fertilization. The same applies for the position of males relative to the eggs during ejaculation.

In most studied fish species, sperm are short-lived, and often exhibit motility for less than a minute. These results which are based on measurements of sperm motility durations in stripped semen [2,3,7,8] lead to the assumption that fertilization takes place fast or even instantaneous (*i.e. *no sperm mortality during fertilization process; [4,9,10]. As direct evidence for a rapid fertilization of the eggs, Fauvel *et al. *[11] showed that seabass (*Dicentrarchus labrax*) sperm exponentially loose their ability to fertilize an egg within a minute after activation. The quick decrease of available gametes in a turbulent environment is proposed as ultimate cause for the evolution of many but short-lived sperm and quick fertilization. Simultaneous gamete release and close proximity between males and females may be further adaptations to the special situation of externally fertilizing fish [12].

In a guarding-sneaker system, sneaker males may not always be able to spawn synchronously with the female and/or in closest proximity to it. Consequently, the sperm competition raffle may often be loaded for a sneaker. If fertilization starts at the moment of sperm release, a sneaking male's success in sperm competition will be a function of the interval in sperm release or the distance to the eggs. In the rainbow trout (*Oncorhynchus mykiss*) the first male sired over 75% of the eggs when sperm of four males were added in 30s intervals [13]. In atlantic salmon (*Salmo salar*) a time difference of only 3s in spawning revealed first male precedence of over 80% [14].

During the breeding season, males of the three-spined stickleback occupy a territory in which they build a tunnel-shaped nest. After male courtship, females lay a clutch of eggs (40–295 eggs: [15]) into the nest. When the female has left the nest or has been chased away after spawning, the male creeps through it and fertilizes the eggs. Neighbouring territorial males may attempt to steal fertilizations by spawning in the foreign nest thus causing sperm competition. In most cases, sneakers ejaculate immediately after the owner [16-18]. The time interval between the guarding and sneaking male in observed sneakings in the lab was usually less than a minute (MZ, pers. obs.). If fertilization occurs as fast as suggested for externally fertilizing fish, every time unit may load the fertilization raffle for the second male. Nevertheless, field data show that sneaking sticklebacks are able to fertilize a huge proportion of a clutch [19]. It is unknown, whether sneakers somehow compensate the load of the raffle (e.g. by an increased ejaculate size), or whether the raffle is not as loaded as it is assumed.

Males and females may have different interests with regard to the speed that eggs are fertilized causing sexual conflict [20] over fertilization time. Males benefit from instantaneous fertilization especially when there is a risk of sperm competition. Females on the contrary may benefit from multiple paternity [21,22] and may therefore aim at prolonged fertilization opportunities for their eggs.

The aim of the present study was to assess the time needed by stickleback sperm to fertilize eggs. Using an *in vitro *fertilization technique, we added freshly gained sperm to eggs and stopped the fertilization process after different time intervals by killing the sperm in two different ways.

## Results

### Main experiment

Males' standard body size averaged 5.67 cm ± 0.10 (SD). Sperm store of the males (*i.e. *the number of sperm in their testes) ranged from 11.2 × 10^7 ^up to 21.2 × 10^7 ^(median: 17.4 × 10^7^). The volume of the sperm suspension containing 25 × 10^6 ^sperm used to fertilize the eggs thus varied in inverse order between 44.64 μl and 23.61 μl (median: 28.7 μl) among replicates. However, the volume of sperm suspension used did not correlate significantly with the fertilization rate in any treatment (Spearman rank correlations, all N = 9, fertilization times of 30s, 120s, 300s, and 600s, P = 0.49, 0.70, 0.21, and 0.72, respectively), nor did the volume per egg (Spearman rank correlations, all N = 9, fertilization times of 30s, 120s, 300s, and 600s, P = 0.62, 0.61, 0.73, and 0.78, respectively).

**Figure 1:**
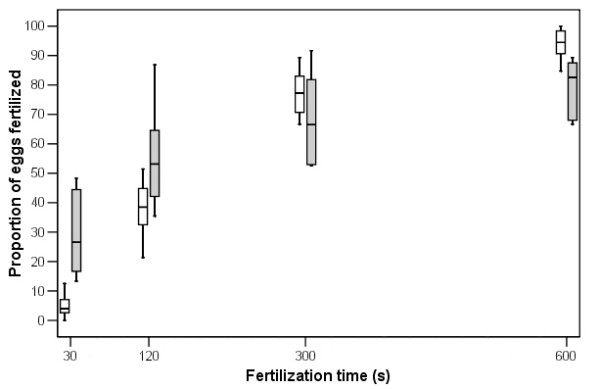
Percentage of fertilized eggs (median, quartiles, range) for the four different fertilization durations. Fertilization was stopped using sparkling mineral water in the main experiment (white bars, N = 9 females), and by washing away the mucus that surrounds the eggs in the control experiment (grey bars, N = 6 females).

The time span in which sperm could fertilize the eggs did significantly affect the fertilization rate (Fig. 1; Friedman-2-way-analysis of variance, N = 9, k = 4, chi-square = 27.00, P < 0.001). 30 seconds after sperm were added, only 4.1% of the eggs (median; range: 0 – 12.5%) were fertilized, which significantly differs from 0 (8 replicates with some fertilizations: Wilcoxon signed-rank test, N = 8, T = 0, P = 0.01). Ten minutes after initiation, fertilization was still not complete in 7 replicates (median of all 9 replicates: 94.4 %; range: 84.75 – 100%; Wilcoxon signed-rank test, N = 7, T = 0, P = 0.02). Thus, fertilization started within the first 30s after addition of sperm, significantly increased with time, but did not reach 100% after 600s.

The number of eggs was not significantly different among the four treatments (*i.e. *different fertilization times); Friedman-2-way-analysis of variance, N = 9, k = 4, chi-square = 5.00, P = 0.17. Mean clutch size was 225.8 (± 71.7 SD), resulting in a mean number of eggs per treatment of 56.4 (± 19.9 SD). The fertilization rate did not correlate significantly with the number of eggs to fertilize in any of the treatments (Spearman rank correlations, all N = 9, fertilization times of 30s, 120s, 300s, and 600s, P = 0.78, 0.36, 0.24, and 0.32, respectively).

### Control experiment

The results of the control experiment were comparable to those of the main experiment. Also here we tested the same four different fertilization times. Males' standard body size averaged 5.00 cm ± 0.15 (SD). The right testis of the six control males contained a median number of 33 × 10^6 ^sperm (range 4.1 × 10^6 ^– 257.5 × 10^6^). The 55 μl of sperm suspension of the left testes used to fertilize the eggs contained thus approximately 8.3 × 10^6 ^sperm (median; range 1.0 × 10^6 ^– 64.4 × 10^6^). The number of sperm per egg thus varied accordingly between 0.43 × 10^5 ^and 11.40 × 10^5 ^among males and treatments (median 3.28 × 10^5^). However, variation was mainly between males as clutches had been divided in approximately four equal portions for the treatments. Thus number of sperm per egg was similar among treatments (Friedman-2-way-analysis of variance, N = 6, k = 4, chi-square = 4.53, P = 0.21). Both the number of sperm per treatment and the number of sperm per egg did not significantly correlate with fertilization rate (Spearman rank correlations, all N = 6, fertilization times of 30s, 120s, 300s, and 600s; sperm per treatment: P = 0.11, 0.40, 0.79, and 0.40, respectively; sperm per egg: P = 0.16, 0.40, 0.54, and 0.87, respectively). In the main experiment the number of sperm per egg was comparable to that in the control experiment (median 4.35 × 10^5^; Mann-Whitney U test, N_1 _= 6, N_2 _= 9, U = 25, 21, 20, and 24, P = 0.86, 0.53, 0.46, and 0.78 for the 30s, 120s, 300s, and 600s treatments, respectively) but the variation was smaller (range 2.53 × 10^5 ^– 10.87 × 10^5^).

Like in the main experiment there was a significant effect of the time period allowed to fertilize the eggs and the fertilization rate in the control experiment (Fig. 1; Friedman-2-way-analysis of variance, N = 6, k = 4, chi-square = 14.00, P = 0.003). Although the change in fertilization rate over available time was qualitatively similar in both experiments, the fertilization rates between the main and control experiments were significantly different for the 30s, 120s, and 600s treatments (Mann-Whitney U test, N_1 _= 6, N_2 _= 9, U = 0, 8, and 4, P < 0.001, P = 0.026, P = 0.005 for the 30s, 120s, and 600s treatments, respectively) but not for the 300s treatment (Mann-Whitney U test, N_1 _= 6, N_2 _= 9, U = 22, P = 0.61).

The mean clutch size (± SD) of 147.5 ± 60.9 was divided into four portion of 36.9 ± 15.3. The portions were similar among treatments (Friedman-2-way-analysis of variance, N = 6, k = 4, chi-square = 4.53, P = 0.21). There existed no significant correlation between fertilization rate and egg number in any of the treatments (Spearman rank correlations, all N = 6, fertilization times of 30s, 120s, 300s, and 600s, P = 0.54, 0.62, 0.87, and 0.40, respectively).

### Fertilization functions

**Figure 2:**
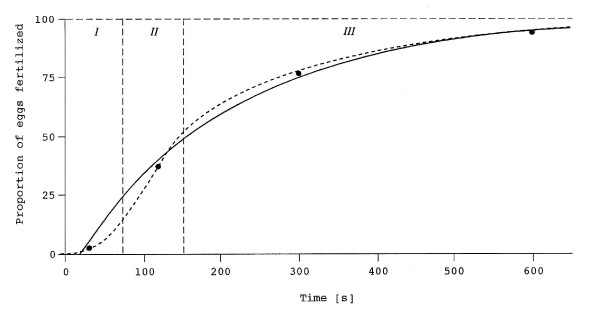
Potential fertilization curves based on the medians (dots) of the experimental fertilization durations (see Fig. 1 white bars). The saturation curve (solid line) assumes instantaneous activation of the sperm after ejaculation, whereas the sigmoid curve (dashed line) assumes that sperm motility starts continuously after sperm release. For a description of the three phases* I – III*, see the text.

Given that all sperm are activated instantaneously after ejaculation and that sperm are present in excess, fertilization by time is expected to follow a saturation curve. Based on the median fertilization rates in the fertilization time treatments of the main experiment (which used sparkling mineral water to kill the spermatozoa), we computed a relationship between fertilization rate and time (Fig. 2; solid line). The resulting equation was: fert. rate = 1 - e^(0.0921 - 0.005*time)^. In this relationship, 50% of the eggs are fertilized 157s after addition of semen, the x-axis intercept is 18.4s.

Alternatively, if sperm motility starts continuously after sperm release, an asymmetric sigmoid function could describe the median fertilization rate over time (Fig. 2; dashed line). This curve is characterized by three phases. The first phase is described by an exponential growth function starting at x = 0 (Fig. 2; segment *I*). The last phase is described by a saturation curve which approaches 1 (segment *III*). The middle phase which is the combination of these two functions may be approximately linear (segment *II*). The time after which 50% of the eggs are fertilized will be very similar in the sigmoid curve and the saturation function, as well as the characteristics of both at more advanced times after sperm release. The main difference between the two functions occurs at the short fertilization times (Fig 2; segment *I*).

Both functions converge 1, and thus assume that fertilization would be complete in the *in vitro *fertilization method.

## Discussion

This study provides evidence that fertilization of an egg clutch in sticklebacks is taking minutes rather than seconds (Fig. 1). This is exceptional if compared to fertilization durations of other teleost fishes [13,14] and contradicts the general assumption that fertilization is very fast in teleost fishes [4,9,10]. However, this assumption is mainly based on the generally very short motility durations of fish sperm [2,3].

For sticklebacks, De Fraipont *et al. *[7] reported sperm motility durations of 300–400s. These data would fit our results. Still, the method used to estimate the motility duration in the mentioned study is likely to produce artefacts due to insufficient dilution of semen [23]. Personal observations (MZ) indicate that sperm are only motile for seconds in our study populations of sticklebacks. Also in a Swedish freshwater stickleback population the mean life span of sperm in freshwater was 30s with single sperm surviving up to 60s [24]. A similar life span in freshwater was measured for sperm of a brackish water population, while sperm from a seawater population was immotile in freshwater [24]. Sperm of all three Swedish populations had much longer life spans (up to 270 min) in brackish water. The longest life spans, except for sperm from the seawater population, were achieved in water with ovarian fluid. In freshwater with ovarian fluid sperm from freshwater fish had a mean life span of incredible 245 min with extreme values of 420 min [24]. Thus the mucus that surrounds the egg clutch will have provided the conditions that enabled the slow fertilization in our experiments. Sperm motility does not seem to be constricted by the viscosity of the mucus [24]. Le Comber *et al. *[25] measured that sperm of English three-spined sticklebacks were able to fertilize eggs for up to 15–20 minutes post ejaculation in fresh water although most measures of sperm activity declined rapidly in the first five minutes. The reduction in sperm motility could be reversed by an increase in osmolality [25]. However, experiments of Elofsson [26] with natural and artificial ovarian fluids suggest that it is rather the ionic than the osmotic effect that prolongs motility in stickleback sperm.

Why does fertilization take that long in sticklebacks? From the male's point of view, fertilization should be quick and instantaneous thereby maximizing fertilization success while minimizing the risk of sperm mortality and sperm competition. The female's point of view is likely to be different. Stickleback males have a limited sperm store [27]. Females cannot run the risk of incomplete fertilization because unfertilised eggs are attacked by fungi within short time and can infect neighbouring healthy eggs. Therefore, a complete fertilization of the eggs may be crucial for successful female reproduction. To keep sperm motile for a long time in the mucus might be an evolutionary adaptation by the females to guarantee complete fertilization.

Additionally, by keeping sperm alive for a long period of time females may provoke sperm competition. It pays for sneaker males to engage in fertilization even if there is a considerable time delay with the ejaculation of the nest owner. Females may benefit from multiple mating in a number of ways. Material benefits are unlikely in the stickleback system, but polyandry may lower the probability of mating with genetically incompatible, inferior or infertile mates as well as increasing next-generation genetic diversity and mean offspring fitness [21,22]. Because the genetic interests of males and females diverge, there exists a sexual conflict over fertilization rate [20,28,29]. Models of sexual conflict predict that females will frequently win the co-evolutionary arms race with males [28] which is apparently the case in sexual conflict over fertilization rate in sticklebacks. It therefore appears that the females have an evolutionary net benefit from provoking sperm competition, even if multiple paternity may have a negative effect on the amount of parental investment a male is willing to invest into a current brood [30].

In our experiments, even after 600s, not all the eggs were fertilized. There may be several reasons for this finding. First, complete fertilization may take longer than 10 min. Second, the *in vitro *fertilization method was not able to fertilize all eggs, or third, incomplete fertilization is a natural phenomenon due to low quality gametes or sperm limitation. Observations of unfertilized eggs in the field are relatively rare (TCMB, pers. obs.), because unfertilized eggs are likely removed by the caring male. *In vitro *fertilization protocols for fish report fertilization rates ranging between 50% to above 85% [11,31-34], but 100% fertilization seems difficult to reach *in vitro*. It is therefore plausible that an artificial situation causes a decrease in the efficiency of fertilization. In our case this would mean that the maximum fertilization was reached after (or even before) the ten minutes (Fig. 1). Consequently, the computed functions that both converge 1 (Fig. 2) would be to some extent misleading. However, these functions should not reveal a precise equation between time and fertilization rate, but visualize the kind of relationship between them. Even if the maximum fertilization was 94% instead of 100%, the basic shape of the curves does not change. We have shown two possible developments of fertilization in time. The main differences between the two curves (*i.e. *saturation and sigmoid curve) are within the first 150s. Thus the form of the curve in the first 150s after fertilization may be crucial when it comes to estimate a sneaker's fertilization success depending on time delay in ejaculation.

Nest-guarding and sneaker sticklebacks sequentially ejaculate their sperm on the available eggs. Nests often contain considerable proportions of eggs that are not fertilized by the nest owner [19,35]. However, the effects of the time difference between the ejaculations remain unknown. Our results suggest that sneakers could fertilize a considerable proportion of eggs despite the time difference they usually have. A possible disadvantage of sneakers because they usually have not the first opportunity to fertilize egg may be compensated by advantages in sperm quality and/or quantity. The risk of sperm competition may therefore not depend on how long a nest owner can physically prevent the sneaker to enter the nest, but categorical whether sneaking will occur or not. Thus, the costs of sneaking may well lead to adaptations to sperm competition in traits like ejaculate size or gamete qualities. One of us (MZ unpublished data) did preliminary quantifications of the time interval between ejaculations of nest owners and sneakers by observing sneakings in a large aquarium containing two territories after introduction of a gravid female. Paternity of the eggs was analysed using microsatellites [19]. Six sneakings were quantified: one sneaker managed to ejaculate shortly before the nest owner, the other sneaking events occurred with a time delay of up to 14 seconds after creeping through the nest of the owner. Although nest owners' sperm had a headstart of up to 14 seconds on sneakers' sperm, all sneakings resulted in lost paternity of the nest owner (range 10.5 – 77.4% of the eggs fertilized by sneakers).

Compared to the *in vitro *protocol for sticklebacks proposed by Barber *et al. *[33,34] that got a mean fertilization rate of 53%, our method clearly improves the state of the art (median after 10 min: 94% in the main experiment and 83% in the control experiment). Due to the differences in sperm concentration between males, different volumes of the sperm suspensions were used to fertilize the eggs in the main experiment. The different volumes used to fertilize did not show any significant correlation with fertilization rate in any treatment. On the other hand, dilution of semen is in many fish the trigger to initiate motility due to changes in osmolality [36-38]. The isotonic medium used for the short-term storage of sperm in our study [11], even though keeping sperm fertile for hours, does not completely inhibit motility (MZ pers. obs.). A small percentage of sperm remains still motile. Different dilutions of the sperm suspension may lead to differences in this motility and consequently in fertilization capability between males.

Mineral water did very efficiently kill sperm. Most likely, the lack of oxygen due to the high partial pressure of CO_2 _caused this effect. However, if CO_2 _also kills freshly fertilized eggs, then these ill-affected eggs cannot be distinguished from unfertilized eggs. A combination of this negative effect of CO_2 _on freshly fertilized eggs and instantaneous fertilization might result in a change in fertilization rate over available fertilization time similar to that of the main experiment. The control experiment shows that this scenario does apparently not apply: a qualitatively similar change of fertilization rate to that in the main experiment was obtained. We cannot completely rule out some negative effects of sparkling mineral water in the treatments with the smallest available periods for fertilization because the fertilization rates in the 30s and 120s treatments of the control experiment were significantly greater than those in the main experiment. Yet an effect of sparkling mineral water seems unlikely as the longer treatments did not show such an effect or even an effect in the other direction. Other differences between the control and main experiment may have effected variation in fertilization rates such as differences in sperm quality due to age differences, or in sperm quantity due to different dilution methods in both experiments. Also washing away the mucus from the eggs in the control experiment may be less efficient at stopping fertilizations or less precise at stopping fertilization at a particular moment of time.

## Conclusion

In conclusion, this study reports an *in vitro *fertilization protocol for an externally fertilizing fish which produces high fertilization rates. In the experiments, fertilizations were stopped after fixed periods of time using two different methods. Compared to other teleosts, fertilization takes exceptionally long time in the three-spined stickleback. This may have resulted from sexual conflict over fertilization rate. Sneakers thus seem to have good chances in obtaining shared paternity of the clutch of eggs, even though the exact interaction of time and fertilization remains to be clarified in such a sperm competition situation. When time is not very costly, strategic investment in the number of ejaculated sperm of competing males could be expected.

## Methods

### Fish

Three-spined sticklebacks (*Gasterosteus aculeatus*) were collected at the end of March during the 2001 spring migration on the island of Texel (The Netherlands) and transported to the University of Bonn (Germany) on the same day. The fish (c. 700) were kept in two mixed-sex storage tanks of about 750 l and supplied with running tap-water keeping the temperature between 16°C and 18°C. From the beginning of June, fish were housed in mixed-sex groups of 18 individuals in aerated and filtrated 80 l aquaria in a climatized room (temperature 17 ± 1°C, 16:8 h light:dark). Fish were fed to satiation with frozen chironomid larvae. The tanks were illuminated by fluorescent lamps (Osram, 36 W) mounted 25 cm above the water level.

The main experiment was performed in September 2001 using nine pairs of reproductively active individuals, that is, males which had developed conspicuous breeding coloration and ripe females that were ready to spawn as judged by the extension of their belly and the dilatation of their genital opening.

### Main experiment

For each trial, a reproductive male and a ripe female were used. Standard body size and mass of both were measured before the experiment. Female's body mass was reassessed after the experiment to estimate egg mass. The male was quickly sacrificed by decapitation, the testes were carefully dissected, and put into an Eppendorff tube containing 200 μl isotonic medium after Fauvel *et al. *[11]. The mass of the testes was weighed with a Sartorius 2004MP balance.

The sperm store in the male's testes was assessed with a Neubauer haemocytometer chamber, following the protocol given in Zbinden *et al. *[27,39]. Testes were homogenised in the Eppendorff tube with a small pestle. In order to avoid pipetting fragments of the testes skin afterwards, the resulting suspension containing the sperm was shortly vortexed and centrifuged (Rotilabo-Mini-Centrifuge, 6000 rpm). 10 μl of the supernatant were then diluted in 190 μl of a 4% formalin solution and vortexed again. One Neubauer haemocytometer chamber was then filled with a 12 μl sample of this solution. After 5 min the number of sperm was counted in 64 cells (2.5 × 10^-4 ^μl each) of the chamber. The total number of sperm (*S*_H_) in the male's testes was calculated as: (*S*_H_) = {(mean no. of sperm per cell) × 400000/10 μl (analyzed volume of the initial suspension)} × 200 (volume of the initial suspension). Based on this estimate, we calculated the volume of the suspension containing 25 × 10^6 ^sperm. This volume was later used to fertilize the eggs.

The egg clutch from the female was stripped into a moistened petri dish by gently squeezing the abdomen with thumb and forefinger. Then, the clutch was carefully and randomly divided into four portions of approximately the same size using fine tweezers. The portions were moved into watch-glasses containing 2000 μl of water. Using a pipette, 25 × 10^6 ^sperm were then distributed over each of the portions. The fertilization of the eggs in the four portions was stopped either after 30, 120, 300 or 600 seconds. This was done by pipetting 1000 μl of fresh commercial sparkling mineral water (Thalquell natural mineral water: Na^+^: 172 mgl^-1^; K^+ ^9.3 mgl^-1^; Mg^+^: 78 mgl^-1^; Ca^2+^: 149 mgl^-1^; Cl^-^: 6.7 mgl^-1^; SO_4 _^2-^: 12.7 mgl^-1^; HCO3^-^: 1281 mgl^-1^: pH: 5.8) into the watch-glass. In preliminary trials, sparkling mineral water gave the best results to kill spermatozoa without harming the eggs. Other methods tested such as the use of acidified water (pH 4 and 3; [40]), as well as a 4% formalin solution (used in fisheries to prevent fungus infection; [41]) did harm the eggs. However, if sparkling mineral water harms freshly fertilized eggs, then these may be indistinguishable from unfertilized eggs. We therefore checked in a control experiment (see below) whether sparkling mineral water is harmless to freshly fertilized eggs. The succession of the four fertilization durations was randomized without repeating an already used sequence.

Ten minutes after fertilization had been stopped for every given treatment, the egg clutch was transferred to a plastic container (20 cl) filled with aerated tap-water. The water was changed at the end of the working day or the next morning. The total number of eggs of each treatment and the number of fertilized/unfertilized eggs was counted 24 h after stopping fertilization using a binocular microscope. Fertilized eggs are clear, show a postfertilization membrane and after 24 h the developing embryo is detectable [42]. Unfertilized eggs were removed and the embryos were reared to hatching.

### Control experiment

In order to rule out possible negative effects of sparkling mineral water on freshly fertilized eggs, a control experiment was added. The control experiment was performed in March 2006 with fish caught in April 2005 from the same population as had been used for the main experiment. Fish were stored in a mixed-sex, outdoor storage tank of 750 l with running tap water and aeration. Two months before the start of the control experiment 100 fish were transferred to two aerated and filtrated 100 l aquaria in a climatized room (temperature 17 ± 1°C, 16:8 h light:dark). Fish were fed to satiation with frozen chironomid larvae and *Artemia*.

The control treatment for which we used six reproductive males and six gravid females was done in the same way as the main experiment with the following exceptions:

i) instead of using sparkling mineral water to stop fertilizations the mucus that surrounds the eggs was washed away with tap water after 30, 120, 300 or 600 sec. To that aim the eggs were put into a metal tea strainer and flushed with a mild stream of tap water of 19 ± 1°C for 45 sec. The greater part of the mucus was already washed away after a few seconds. Once the mucus was completely washed away the eggs hardened. Test trials showed that when immediately after washing fresh sperm was added to the eggs, hardly any egg could be fertilized any more.

ii) instead of using a fixed number of sperm to fertilize the eggs a fixed volume of 55 μl of sperm suspension was used. The sperm suspension was prepared as in the main experiment but with 220 μl instead of 200 μl isotonic medium [11]. For fertilization sperm from the left testis were used. After sperm was added to the eggs the right testis was prepared in 200 μl isotonic medium for sperm counting. In fish of the Texel population sperm number in left and right testes were not significantly different (paired t test, t = 0.90, df = 12, P = 0.39) and correlated well (r_p _= 0.72, N = 12, P = 0.009) (TCMB & M. Hollmann unpublished data).

### Statistical analyses

Analyses were performed using SPSS 12.0 for Windows. Due to the small sample size, non-parametric analyses were used. A non-linear regression was applied to compute a hypothetical function based on four means. Given P values are two-tailed throughout.

## Authors' contributions

TCMB, MZ, and CRL conceived of the study. MZ designed the main experiment which AW performed, TCMB and JGF designed the control experiment which JGF performed. Fish were collected by MZ and JGF. Statistical analyses were done by TCMB, MZ, and AW. TCMB drafted the manuscript and was helped by MZ, AW, and CRL. All authors contributed to the study and approved the final manuscript.
